# P05.58. Yoga is a feasible intervention for veterans with chronic stroke

**DOI:** 10.1186/1472-6882-12-S1-P418

**Published:** 2012-06-12

**Authors:** M Van Puymbroeck, A Schmid, K Miller, N Schalk

**Affiliations:** 1 Indiana University, Bloomington, USA; 2 Roudebush VAMC/ Indiana University, Indianapolis, USA; 3 Heartland Yoga, Indianapolis, USA

## Purpose

Due to the machismo environment that often pervades the military, and thus the patients at Veterans Affairs Medical Centers, we wanted to explore if a yoga intervention was feasible with this population.

## Methods

Focus groups were conducted after the completion of four waves of an 8-week yoga intervention. Twenty-six veterans participated in the focus groups. The focus groups ranged in length from 30 to 90 minutes, and were digitally recorded, and transcribed verbatim. Constant comparison analyses were used to determine initial and emerging themes.

## Results

Four primary themes emerged from the data. Related to preconceived notions, veterans identified that they did not know what to expect, in part because stretching activities seemed too simple to help. While we called this “balance exercise,” veterans had mixed feelings about calling this yoga. All were more accepting of this by the end of the study. However, several, in addition to saying they wouldn’t know what to expect from yoga, said “men don’t do yoga” while another compared it to voodoo. The veterans identified that benefits from participation in a group post-stroke yoga intervention included: acknowledgement of the idea that while there was no pain and the yoga was gentle, it was still effective and powerful; the yoga activities could be done at home alone; and it filled the gap after rehabilitation. Finally, we learned that when working with veterans who had seen combat, there were some important considerations when designing an intervention. These included making sure the participants were not seated with their backs to the door, and introducing all helpers at the beginning of each session, and identifying their role.

## Conclusion

Yoga is a feasible intervention for veterans who have had a stroke. However, there are important considerations that need to be taken into account when planning a study for veterans.

